# Effects of Dante’s Poetry on Emotional Arousal of Healthy Subjects and Patients with Spinal Cord Injury: A Pilot Study

**DOI:** 10.3390/bs16081323

**Published:** 2026-08-03

**Authors:** Claudia Salera, Maria Teresa Perrotta, Franco Marinozzi, Liliana Baleca, Maria Pia Lucia, Paola Coiro, Giada Serratore, Federica Tamburella, Marco Iosa

**Affiliations:** 1 Department of Psychology, Sapienza University of Rome, 00185 Rome, Italy; claudia.salera@uniroma1.it (C.S.); mariapia.lucia@uniroma1.it (M.P.L.); 2 Department of Mechanical and Aerospace Engineering, Sapienza University of Rome, 00185 Rome, Italy; perrotta.1750597@studenti.uniroma1.it (M.T.P.); franco.marinozzi@uniroma1.it (F.M.); 3 LIFE—Institute for Research and Treatment, Santa Lucia IRCCS, 00179 Rome, Italy; l.baleca@hsantalucia.it (L.B.); p.coiro@hsantalucia.it (P.C.); g.serratore@hsantalucia.it (G.S.); f.tamburella@unilink.it (F.T.); 4 Department of Life Sciences, Health and Health Professions, Link Campus University of Rome, 00165 Rome, Italy

**Keywords:** emotion, neuroaesthetics, poetry, rehabilitation, cultural well-being

## Abstract

Previous studies have investigated the effects of Dante’s poetry on emotional arousal and cognitive workload in healthy participants by comparing excerpts from the *Inferno* and *Paradiso* of the *Divina Commedia*. In this pilot study, conducted in a hospital setting, we measured electrodermal activity, an index of emotional arousal, in healthy subjects and neurological patients with spinal cord injury while they listened to four different audio recordings: Dante’s *Inferno*, Dante’s *Paradiso*, Machiavelli’s *Il Principe*, and a newspaper article about the anniversary of Dante’s death. We found significantly higher emotional arousal in both healthy subjects and patients when listening to Dante’s *Paradiso*, than when listening to Machiavelli’s *Il Principe* or the newspaper article. In contrast, no significant difference emerged between the *Paradiso* and the *Inferno*. These findings were supported by higher scores assigned to Dante’s recordings on the Beauty Assessment Scale. In this explicit assessment, participants preferred Dante’s *Inferno*. Finally, the results showed a reduction in negative affect after the entire task. In conclusion, the electrodermal activity results, combined with the explicit aesthetic scores in this pilot study, suggest that Dante’s poetry may enhance emotional arousal in hospital workers, caregivers, and patients hospitalized for rehabilitation.

## 1. Introduction

For centuries, the study of poetry has been regarded as part of literature, but more recently a new branch of neuroaesthetics, called neurocognitive poetics, has emerged. This transdisciplinary field empirically investigates poetic literature, including its neural underpinnings and psychological effects (Jacobs, 2015).

Unlike prose, poetry is often characterized by rhythmic and metrical features that may substantially contribute to persuading, moving, and pleasing an audience. These recurring patterns may enhance perceptual fluency and, consequently, aesthetic appreciation, according to the “cognitive fluency theory” (Obermeier et al., 2016). On the other hand, poetry is often characterized by non-literal language, rich in metaphors and other rhetorical figures, which can reduce the ease of cognitive processing because poetry may require greater attention and mental effort (Sun et al., 2022). Some studies have shown that poetic language may engage people in an “embodied simulation” (Gibbs & Okonski, 2018) and promote a flexible adaptation of semantic memory to imagine and construct novel figures of speech (Benedek et al., 2014).

Poetry has also shown potential in promoting patients’ well-being. In fact, exposure to audio recordings of poetry has been associated with positive mental health outcomes, including reductions in stress, anxiety, and depression; improvements in quality of life; and the promotion of hope among patients with cancer (Arruda et al., 2016; Gozashti et al., 2017). Literature therapy (poetry and story therapy) has proven to be an effective assistive tool for treating neurosis as well as emotional or behavioral disorders in patients with stroke, serving as a useful auxiliary tool for the rehabilitation process (Eum & Yim, 2015). Qualitative findings suggest that poetry has positive effects on the renegotiation of identity and the sense of self after trauma through a metaphorical lens, providing a mechanism for reframing illness narratives into new narrative identities (Hoepner et al., 2025). Most of these effects have been qualitatively assessed using validated questionnaires and scales.

A recent study quantitatively assessed the cognitive and emotional impact of selected passages from the *Divina Commedia* on experts and non-experts (Cartocci et al., 2021). The *Divina Commedia* is one of the most important, famous, and widely studied literary works worldwide, particularly in Italy, and its meter and rhymes may promote processing fluency according to neuroaesthetic theories (Cartocci et al., 2021). The poem is composed of three canticles, each characterized by different themes: *Inferno* (Hell), focused on sins; *Purgatorio* (Purgatory), focused on redemption; and *Paradiso* (Paradise), focused on beatitude. Interestingly, Cartocci et al. (2021) found greater emotional engagement (assessed through galvanic skin response) among non-experts compared to experts. Experts, however, showed greater cognitive workload, an index of frontal cortex activity measured using electroencephalography. Moreover, non-experts exhibited a higher emotional response and greater cognitive effort when listening to the verses of *Paradiso* compared to those of *Inferno*.

Although emotional engagement has been instrumentally measured in healthy subjects (Cartocci et al., 2021), the potential impact of Dante’s poetry on people in hospital settings, including workers, caregivers and patients with a traumatic neurological disease, has never been investigated. The inclusion of individuals with spinal cord injury was motivated by the aim of assessing whether emotional arousal in response to aesthetic auditory stimuli observed in healthy participants is also present in a clinical population characterized by altered sensorimotor and affective processing (Anderson, 2004; Chatterjee & Vartanian, 2014). Emotional activation is usually considered a positive factor for clinical prognosis (Pruneti et al., 2023), because conditions characterized by autonomic hypoactivity are associated with increased depressive symptom intensity and a worse prognosis due to reduced motivation and participation (Mori et al., 2016). Conversely, emotional arousal may reduce depressive symptoms (such as anhedonia and alexithymia), which have been identified as key psychological factors in patients with spinal cord injury (Mascanzoni et al., 2024). Poetry may also help to alleviate stress and burden among counselors and caregivers (Boone & Castillo, 2008). Importantly, this line of research should be understood as an exploratory psychophysiological investigation of emotional responses to aesthetic auditory stimuli in a clinical environment, rather than as a therapeutic intervention. The aim of this study was to investigate emotional arousal, assessed through electrodermal activity, in hospital workers, caregivers and patients hospitalized for rehabilitation, with the expectation of the highest emotional arousal for Dante’s poetry.

## 2. Materials and Methods

### 2.1. Participants

We enrolled and tested 28 participants in a hospital setting (mean age: 51.2 ± 19.7 years, 12 females and 16 males). Eight participants were patients with spinal cord injury who were hospitalized for rehabilitation (2 females, 6 males; mean age 65 ± 18 years); nine were caregivers (5 females, 4 males; mean age 63 ± 7 years); and eleven were hospital workers and trainees (5 females, 6 males; mean age 31 ± 8 years). All participants completed the same experimental protocol, consisting of a single audio listening session conducted in the hospital. All participants were recruited voluntarily and provided informed consent. The protocol was approved by the independent Ethics Committee of Lazio Area 5 (approval code: 154/SL/24).

### 2.2. Stimuli

Four auditory stimuli were presented, all recorded by the same narrator voice: excerpts from Dante’s *Paradiso* (Canto XXXIII) and *Inferno* (Canto V), Chapters 1–2 of Machiavelli’s *Il Principe* (The Prince), and a newspaper article related to the anniversary of Dante’s death. The latter two stimuli served as control stimuli: the newspaper controlled for content, whereas *Il Principe* controlled for the use of archaic Italian. Each auditory stimulus lasted 150 s. Two fixed sequences of audio stimuli were counterbalanced across participants to minimize order effects: (1) *Paradiso*, *Il Principe*, *Inferno*, newspaper article; and (2) the reverse order.

### 2.3. Procedure

Experiments were conducted between October 2025 and March 2026. Participants were informed about the recording procedures but not about the content of the stimuli to avoid expectancy bias. Demographic information (age, gender, years of education) was collected, and affective state was assessed using the Positive and Negative Affect Schedule (PANAS; Watson et al., 1988), both as trait and state measures. Cognitive function was screened using the Mini-Mental State Examination (MMSE). All participants scored above the screening threshold and were therefore included in the study. Physiological signals were recorded using a wearable device (Shimmer3 GSR+). Electrodermal activity (EDA) electrodes were placed on the proximal phalanges of the index and middle fingers of the non-dominant hand. Participants were seated in a quiet environment, instructed to remain still, and wore Bluetooth headphones. The protocol consisted of four experimental recordings, each corresponding to one auditory stimulus and preceded and followed by 5 s of baseline. After each stimulus, participants completed the Beauty Assessment Scale (BAS) (Iosa et al., 2025; validation of the BAS on auditory and poetic stimuli is currently under review), an eight-item scale grounded on four dimensions of beauty: subjective (items 1 and 2), objective (items 3 and 4), emotional (items 5 and 6), and cognitive (items 7 and 8). At the end of the listening task, the PANAS state assessment was repeated, and the artistic interest was assessed using a numeric rating scale (“How much are you interested in art?”; scored from 0 to 10). The experimental protocol was administered by the researchers involved in the present study.

### 2.4. Data Acquisition

The EDA signal was acquired at a sampling frequency of 51.2 Hz (e.g., Tonacci et al., 2019, 2021) with the Shimmer3 GSR+ device. Data were streamed via Bluetooth and recorded with Consensys software (v2.1.2). Signal processing was performed offline in MATLAB using standardized pipelines (MATLAB version: R2026a, Natick, MA, USA: The MathWorks Inc.).

### 2.5. EDA Signal Processing

EDA preprocessing included outlier removal (threshold: mean + 3 standard deviations). Outliers were replaced with local averaging, computed as the mean of the preceding sample and the second subsequent sample, to avoid cases of two consecutive outliers. To remove noise and motion artifacts, EDA signals were low-pass filtered using a 4th-order zero-phase Butterworth filter with a 1 Hz cutoff, following recommendations from previous studies (e.g., Kim et al., 2018; Posada-Quintero & Chon, 2020) and the Shimmer User Guide (rev. 1.13, Shimmer Research, Dublin, Ireland, 2018). To ensure comparability across conditions, all signals were cut to 135 s (6912 samples). The first 5 s of each recording were removed and used for baseline correction. To account for inter-individual differences in baseline EDA levels, data were normalized within participants using Z-scores computed across the four experimental conditions.

### 2.6. Statistical Analysis

Means and standard deviations of the data were computed, and then transformed into t-points (50 + 10*Z-score) to normalize the data across the four conditions within each subject. A Mixed Analysis of Variance (ANOVA) was performed for EDA and BAS t-scores with two within-subject factors: Dante’s poetry (Paradiso and Inferno vs. Principe and Article) and Cognitive Effort (CE: Paradiso and Machiavelli vs. Inferno and Article). As previously reported for subjects who were non-experts in the literature, listening to Paradiso XXXIII required greater cognitive effort than Inferno V (Cartocci et al., 2021). Given the reduced sample size, between-subject factors were limited to Group (hospital workers, caregivers, and patients) and Order of stimulus presentation (sequence 1 or 2, with the second being the reverse of the first). Post hoc tests were performed using Tukey correction on *p*-values. Effect sizes were computed in terms of partial eta squared (η_p_^2^). Pearson’s coefficient (R) was used to assess the correlation between variables. PANAS scores were not normalized, and the pre- vs. post-experiment comparisons were performed using the Wilcoxon rank test. For all analyses, the significance level was set at 0.05. The software used for statistical analysis was Jamovi (The jamovi project, version 2.3.28.0).

## 3. Results

**Figure 1:**
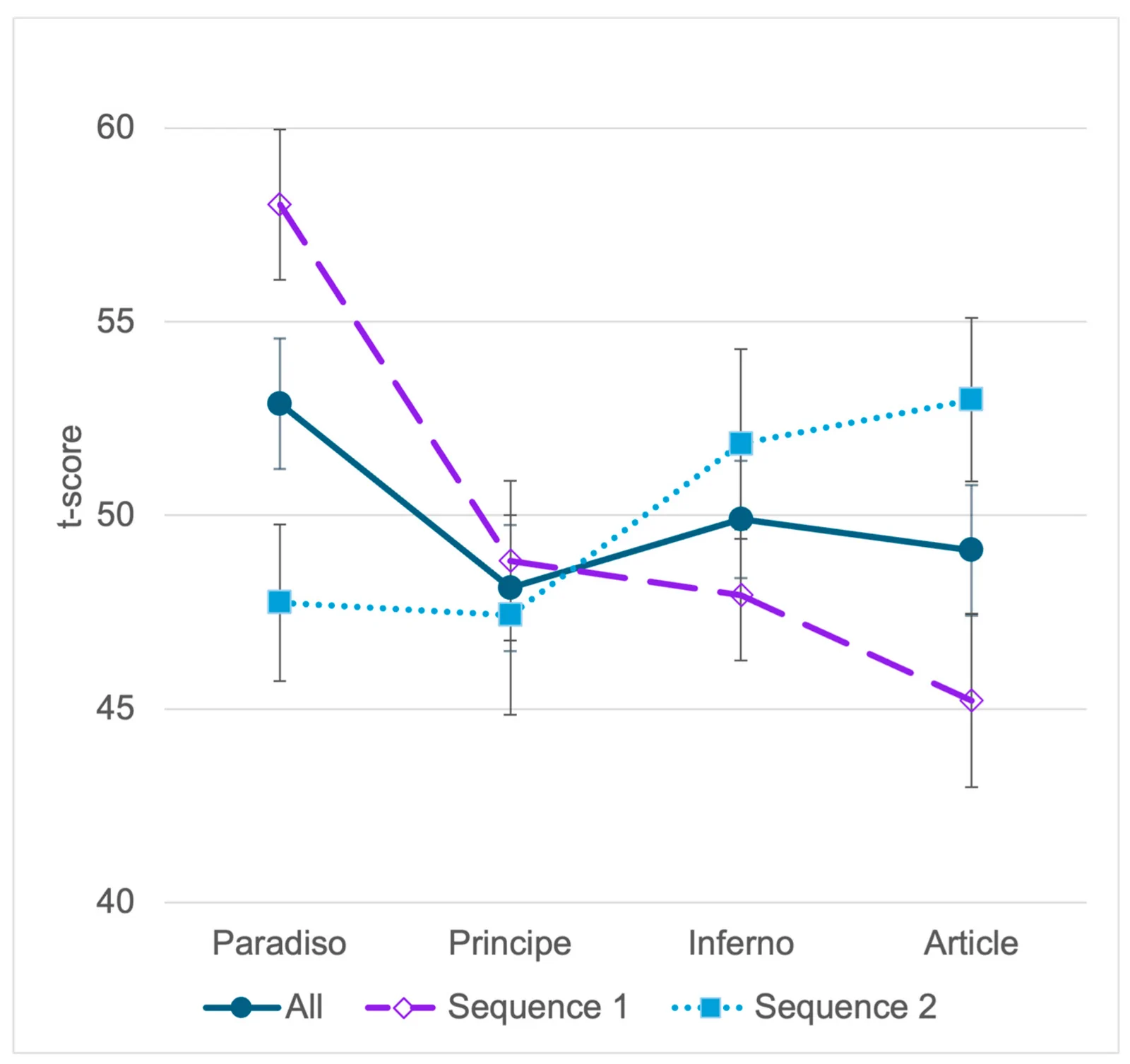
Means and standard errors of the t-scores for EDA data (in blue for all subjects, violet dotted line and diamonds for those assigned to sequence 1, and light blue dotted line and squares for those assigned to sequence 2).

**Figure 2:**
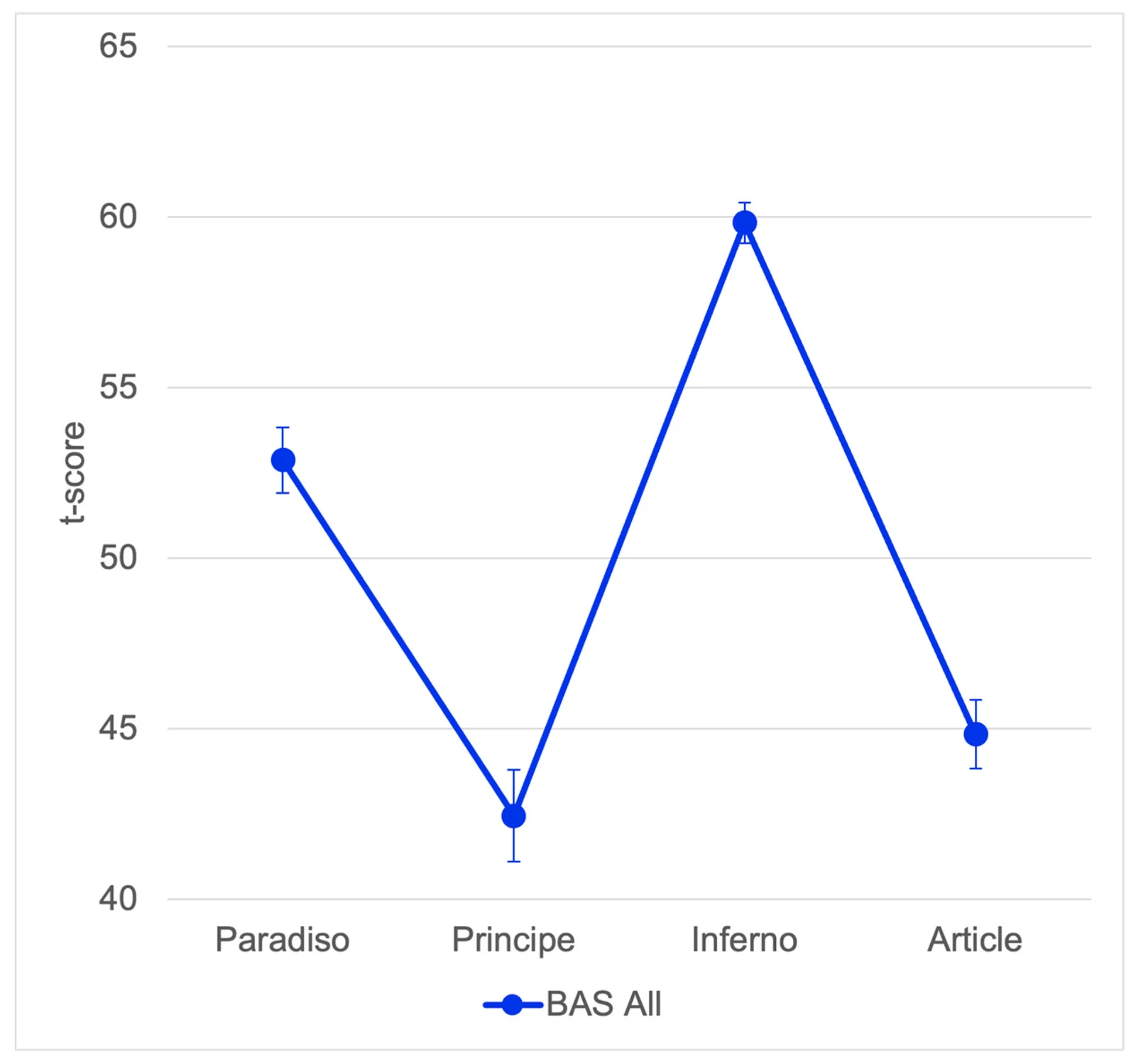
Means and standard errors of the t-scores for the BAS data across conditions.

Figure 1 shows EDA t-scores across the four experimental conditions. Figure 2 shows BAS t-scores across the same conditions. The Mixed ANOVA showed a significant main effect of Dante’s Poetry on EDA t-scores (F(1,22) = 5.586, *p* = 0.027, η_p_^2^ = 0.202). Neither Cognitive Effort (*p* = 0.585) nor Order (*p* = 0.077) had a significant main effect on EDA t-scores. Two significant interactions were found with Poetry. The larger effect size was observed for the Poetry by Group interaction (F(2,22) = 8.758, *p* = 0.002, η_p_^2^ = 0.443). Post hoc analysis showed that EDA t-scores during Poetry listening were higher in hospital workers (*p* = 0.013) and patients (*p* = 0.026) than in caregivers. Conversely, during Prose listening, EDA t-scores were higher in caregivers than in hospital workers (*p* = 0.013) and patients (*p* = 0.026). The second significant interaction was between Poetry and Order (F(2,22) = 6.123, *p* = 0.022, η_p_^2^ = 0.218). Post hoc analysis showed that the difference between Poetry and Prose was greater in sequence 1 (*p* = 0.012).

A secondary-level analysis showed higher EDA levels for *Paradiso* than for *Il Principe* (*p* = 0.014) and the article (*p* = 0.010). Consistent with the main ANOVA result showing no significant Poetry x Cognitive Effort interaction (*p* = 0.269), no statistically significant differences were found between *Paradiso* and *Inferno* (*p* = 0.128), or in any other pairwise comparisons.

No significant correlations were found between EDA t-scores and age or education. Analyses of raw EDA scores revealed a significant correlation between age and EDA during listening to *Il Principe* (R = 0.531, *p* = 0.004). For the article, the correlation only approached statistical significance (R = 0.369, *p* = 0.053), whereas no significant correlations were found for *Paradiso* (R = 0.227, *p* = 0.246) and *Inferno* (R = 0.279, *p* = 0.151). Neither raw EDA data nor relevant t-scores were significantly correlated with education. When the Mixed ANOVA was applied to BAS t-scores, a significant main effect of Dante’s Poetry was found (F(1,22) = 128.204, *p* < 0.001, η_p_^2^ = 0.854) together with a significant effect of Cognitive Effort (F(1,22) = 15.201, *p* < 0.001, η_p_^2^ = 0.409). As shown in Figure 2, the BAS t-scores were higher for Poetry than for Prose, and the two audio stimuli requiring lower Cognitive Effort (*Inferno* and Article) were preferred over the other two (*Paradiso* and *Principe*).

No significant correlations were found between BAS t-scores and either age or education. A trend was observed between age and BAS scores during *Il Principe* (R = 0.348, *p* = 0.069). Analysing the single domains of the BAS data, a significant effect of condition emerged for *Inferno* (F(3,81) = 3.79, *p* = 0.013, η_p_^2^ = 0.0123), due to higher emotional beauty scores (items 5 and 6 averaged for each participant) compared with cognitive beauty (items 7 and 8 scores averaged for each participant; *p* = 0.002). No differences among the four BAS domains were observed for *Paradiso*.

**Figure 3:**
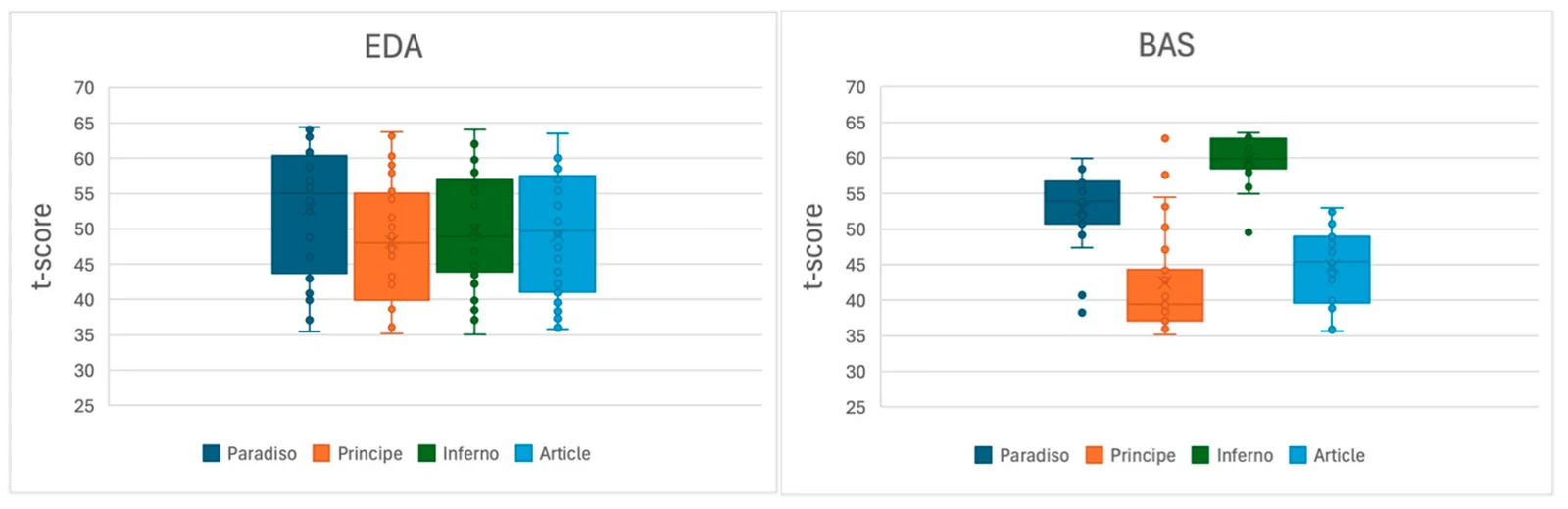
Box-and-whiskers plot showing the median and quartiles of participants’ EDA (on the (**left**)) and BAS (on the (**right**)) t-scores, with dots representing individual points across conditions.

Figure 3 shows participants’ mean EDA and BAS scores as individual points across conditions.

Negative affect scores assessed by the PANAS were significantly reduced after completion of the experiment in the whole sample (*p* < 0.001), whereas positive affect scores did not change significantly (*p* = 0.610). In particular, subjects reported feeling less irritable (1.96 ± 1.29 vs. 1.29 ± 0.76, *p* = 0.010) and less ashamed (1.64 ± 0.83 vs. 1.46 ± 0.84, *p* = 0.007) after the experiment. Although the overall positive affect score did not vary significantly, the score related to feeling inspired increased significantly after the experiment (2.96 ± 1.04 vs. 3.39 ± 1.03, *p* = 0.020).

## 4. Discussion

Results showed that listening to Dante’s excerpts elicited greater emotional arousal and aesthetic appreciation than listening to prose, such as *Il Principe* and the newspaper article. In fact, both EDA and BAS t-scores were influenced by a significant main effect of poetry. For EDA, this effect was greater among hospital workers and patients with spinal cord injury, and in sequence 1 (as reported on the *x*-axis of the figures).

Dante’s poetry is characterized by a tercet structure and a highly regular rhyme scheme, features that may facilitate predictive processing, a mechanism recently associated with aesthetic appreciation (Frascaroli et al., 2024). Building on the study by Cartocci et al. (2021), we compared Dante’s *Paradiso* and *Inferno* with a newspaper article about the anniversary of Dante’s death and with the first two chapters of Machiavelli’s *Il Principe*. The results revealed higher electrodermal activity, particularly while listening to *Paradiso*. Similarly, in Cartocci et al. (2021), *Paradiso* elicited greater emotional arousal and greater cognitive effort.

A progressive reduction in EDA was observed throughout the experiment. We hypothesize that this effect may reflect a progressive habituation or reduced interest, a phenomenon previously reported in the literature (Isen et al., 2013), rather than a reduction in sensor sensitivity. This interpretation is supported by the fact that the recording device was restarted before each trial, and baseline correction was applied to each recording. The reduction in EDA could also be related to progressive relaxation in participants; for this reason, it was important to account for the order of stimulus presentation in the analysis. Moreover, the significant condition-by-order interaction, together with the absence of a significant main effect of order, suggests that the reduction in EDA was stimulus-dependent. It was therefore important to randomize the subjects into two opposite audio-listening sequences. Notably, the higher EDA response associated with *Paradiso* was observed only when this stimulus was presented first (sequence 1), suggesting a novelty or alerting effect related to stimulus position. Nevertheless, the persistence of a significant Condition effect indicates that stimulus content likely contributed to EDA responses.

Age represents another potential confounding factor. Older skin tends to be drier than younger skin, and electrodermal activity generally decreases with age (Barontini et al., 1997; Eisdorfer et al., 1980; Gavazzeni et al., 2008; Kronholm et al., 1993), although mixed results have also been reported (Greene, 1976; Shibagaki et al., 1994; Venables & Mitchell, 1996). In the present study, age-related variability was partially controlled by computing within-subject t-scores. Consistent with this, the significant correlation between EDA and age during listening to *Il Principe* emerged only from the raw data, but not when t-scores were used, possibly suggesting that the association was mainly attributable to inter-individual variability rather than a stimulus-specific effect. However, future studies involving larger samples and sets of stimuli may further clarify this issue.

The explicit assessment of aesthetic appreciation, recorded using the Beauty Assessment Scale, was not affected by the order of presentation. However, it clearly showed higher ratings for the two Dante excerpts than for the control conditions. Interestingly, *Inferno* received higher BAS scores than *Paradiso*, mainly due to increased ratings in the emotional beauty domain. *Paradiso* focuses on the reward for having faced difficulties in aligning the intellectual faculties with a higher moral order. In contrast, *Inferno* is generally more closely related to sin, pain, loss of rational control, and negative affect, although the selected verses from Canto V were also concerned with love and passion (Suurmond, 2026).

Electrodermal activity is positively associated with emotional engagement and arousal, but it does not provide information about emotional valence. Increased EDA may reflect positive emotions such as pleasure, but it can also reflect negative states such as anxiety or stress. Conversely, reduced EDA is usually associated with conditions such as depression and apathy, which are characterized by low arousal (Pop-Jordanova & Pop-Jordanov, 2020). However, after the experiment, all participants reported a reduction in negative affect, as assessed by the PANAS. Specifically, participants reported feeling less irritable and less ashamed, while feelings of inspiration increased, generally suggesting an increase in well-being. As the PANAS was administered only before and after the completion of the entire experimental protocol, it is only possible to hypothesize that the observed reduction in negative affect may reflect the overall experimental context, including but not limited to poetry listening. This assumption would be in line with the results of previous studies showing that poetry may reduce stress, anxiety, and depressive symptoms in patients (Kassab et al., 2026; Arruda et al., 2016; Gozashti et al., 2017) and in hospital workers (Liu et al., 2025). In general, the use of artistic stimuli in hospital settings, even in a single session, was associated with increased motivation and reduced perceived fatigue in patients, a phenomenon known as the Michelangelo effect (Iosa et al., 2021; Tieri et al., 2024). Such findings are particularly relevant for individuals with spinal cord injury, who frequently experience these symptoms (Mascanzoni et al., 2024; Gklantzouni et al., 2025). Psychological interventions are a key aspect of rehabilitation for these patients, and future research should investigate whether carefully selected poetic stimuli may promote emotional engagement while reducing negative feelings in this population.

Our results should be interpreted with caution given the limitations of this pilot study, in particular, the reduced sample size of the patient group and the age disparity between the hospital workers and the other two groups: patients and caregivers. The patient group was limited to eight participants and therefore cannot be considered representative of the broader population of individuals hospitalized for spinal cord injury, given the high variability in severity and symptoms associated with this condition.

Moreover, heterogeneity was present not only between groups but also within each group. However, our aim was to examine individuals in hospital settings, either as patients, caregivers, or professionals. Our protocol did not allow us to separate a general effect of poetry potentially related to increased processing fluency induced by its metrics and rhymes (Cartocci et al., 2021), from a specific effect of Dante’s poetry, although some differences were found, especially for explicit BAS scores, which were higher for Inferno and the newspaper article, two audio stimuli associated with lower cognitive effort. We partially replicated evidence of higher EDA for *Paradiso* than for *Inferno* in non-expert participants, as reported by Cartocci et al. (2021), although in our study this effect depended on the sequence of stimulus presentation. Familiarity with poetry, cultural background, emotional state, medication effects, and the broader rehabilitation context may have affected EDA responses, but were not assessed in this pilot study. We combined electrodermal activity (EDA), a measure of emotional arousal that does not provide information about emotional valence, with Beauty Assessment Scale scores to assess aesthetic appraisal. However, it is difficult to determine whether the observed physiological activation reflected meaningful emotional changes, let alone whether they could be associated with a therapeutic effect. To assess positive and negative affect, we administered the PANAS, but only at the end of the experimental session. Future research should include additional psychophysiological and affective measures to provide a clearer interpretation of our findings.

Finally, we tested subjects in a single session; further studies should investigate the effects of repeated exposure to poetry in hospital settings, possibly with larger samples to reduce the risk of null findings due to low statistical power. Moreover, a control group could be tested during a standardized baseline of equivalent duration to investigate whether the reduction in negative affect is attributable to the participants’ resting-state period or requires exposure to poetic stimuli. Future research should use larger longitudinal, multicenter studies to assess the possible therapeutic effects of poetry and its potential clinical implications, which were not investigated in this pilot study.

In general, our results may provide empirical support for the well-known claim pronounced by the character played by Massimo Troisi in the film “The Postman,” in which he delivers the post to the famous poet Pablo Neruda: “Poetry does not belong to those who write it; it belongs to those who need it.”

In conclusion, this pilot study showed that selected excerpts from Dante’s *Paradiso* increased emotional arousal in a group of people working in, attending, or undergoing rehabilitation in a hospital setting, with some general positive effects on participants’ transient affective state at the end of the experimental procedure. However, these effects must be interpreted cautiously, as they were measured across the entire protocol and included different literary genres.

## Data Availability

Data are available on OSF at https://osf.io/b4nfv (accessed on 29 April 2026).

## Abbreviations

The following abbreviations are used in this manuscript:

PANAS	Positive and Negative Affects Scale
MMSE	Mini-Mental State Examination
EDA	Electrodermal Activity
BAS	Beauty Assessment Scale
ANOVA	Analysis of Variance
